# Development and Validation of the Infection Prevention Appraisal Scale

**DOI:** 10.3390/nursrep13010017

**Published:** 2023-01-31

**Authors:** Maria Lindberg, Magnus Lindberg

**Affiliations:** 1 Faculty of Health and Occupational Studies, Department of Caring Sciences, University of Gävle, 801 76 Gävle, Sweden; 2 Centre for Research and Development, Region Gävleborg/Uppsala University, 801 87 Gävle, Sweden; 3 Department of Public Health and Caring Sciences, Uppsala University, 751 22 Uppsala, Sweden

**Keywords:** behaviour change, classic test theory, content validity index, exploratory factor analysis, infection prevention, organism transmission, scale development, self-efficacy

## Abstract

It has been emphasized that future studies aimed at improving adherence to infection prevention guidelines should focus on beliefs in, e.g., self-efficacy. Reliable situation specific measures are needed to measure the phenomenon of self-efficacy, but there seems to be few valid scales available that are suitable for measuring one’s belief in self-efficacy regarding infection prevention measures. The purpose of the study was to develop a unidimensional appraisal scale to capture nurses’ beliefs in their abilities to practice medical asepsis in care situations. When creating the items, evidence-based guidelines for preventing healthcare-associated infections were used together with Bandura’s guide for constructing self-efficacy scales. Face validity, content validity, and concurrent validity were tested in various samples of the target population. Furthermore, dimensionality was evaluated on data collected from 525 registered nurses and licensed practical nurses recruited from the medical, surgical, and orthopaedic wards of 22 Swedish hospitals. The Infection Prevention Appraisal Scale (IPAS) consists of 14 items. Face and content validity were endorsed by target population representatives. The exploratory factor analysis suggested unidimensionality, and the internal consistency was good (Cronbach’s alpha 0.83). The total scale score correlated with the General Self-Efficacy Scale, as expected, and supported concurrent validity. The Infection Prevention Appraisal Scale demonstrates sound psychometric properties supporting a unidimensional measure of self-efficacy to medical asepsis in care situations.

## 1. Introduction

Infection prevention measures, which prevents or stops the spread of microorganisms, are fundamental in healthcare settings due to their impact on patient and personnel safety. Infection prevention guidelines generally provide recommendations on how to obtain medical asepsis in care situations. For instance, the national evidence-based guidelines described by Loveday et al. [1] incorporate standard infection control principles regarding hospital environmental hygiene, hand hygiene, use of personal protective equipment, safe use and disposal of sharps, and principles of asepsis. Adherence to all infection prevention measures helps to minimize the risk of acquired healthcare-associated infections for patients and personnel. Thus, non-adherence to infection prevention measures constitutes a risk behaviour for organism transmission.

The single most referred to and accepted measure to prevent healthcare-associated infections according to the literature is adherence to hand hygiene. Even so, organism transmission occurs in practice because of inadequate hand disinfection, incorrect use of gloves, and deficient surface and equipment disinfection [2,3,4,5,6]. Studies from all over the world have shown various rates of hand hygiene adherence among nurses. A recent systematic review and meta-analysis focusing on nurses’ hand hygiene adherence in high-income countries show that the adherence rate is 52% [7]. Many interventions with content that includes single or multimodal approaches have been conducted in an effort to improve healthcare practitioners’ adherence to hygiene protocols including hand hygiene and personal protective equipment. Systematic reviews concluded, however, that multimodal interventions have shown small improvements regarding hand hygiene adherence, but it was not possible to give specific endorsements regarding the contents of the interventions or the methods of delivery [8,9]. To gain successful results, interventions should involve a focus on social influence, attitude, self-efficacy, and intention [9]. Many of the recommended infection prevention measures are simple and can be performed to a relatively low cost. However, adherence to such preventions requires accountability from healthcare personnel as well as a behavioural change. Implementing behavioural change is challenging. Jackson et al. [10] concluded that nurses’ authentic infection prevention behaviour rarely coincides with their own perceptions. Deficient knowledge is not the cause of non-adherence. This awareness calls for infection prevention interventions that consider beliefs in, such things as self-efficacy, values, and social understanding.

In an integrative literature review that included six articles, Pereira et al. [11] summarized that self-efficacy is essential for the adherence and performance of hand hygiene practices. Self-efficacy concerns a person’s own belief in his or her own ability to perform a task, which is dependent on the person’s level of self-confidence in performing that specific task. Furthermore, it is about believing that one’s skills are possible to develop by means of personal effort. A high sense of self-efficacy when performing a specific task does not necessarily mean that a person will have the same sense of high self-efficacy when performing another task [12]. That being so, a need to have high, situation-specific self-efficacy is essential to be able to change one’s behaviour [11]. Persons having a high sense of self-efficacy see challenges as something they want to conquer instead of problems they want to avoid [12]. Thus, research focusing on the aspect of self-efficacy in regard to infection prevention measures among healthcare personnel is needed. As there is a lack of validated scales to measure the aspect of self-efficacy in regard to infection prevention measures, our aim was to develop a unidimensional appraisal scale to capture nurses’ beliefs in their abilities to practice medical asepsis in care situations.

## 2. Materials and Methods

**Figure 1:**
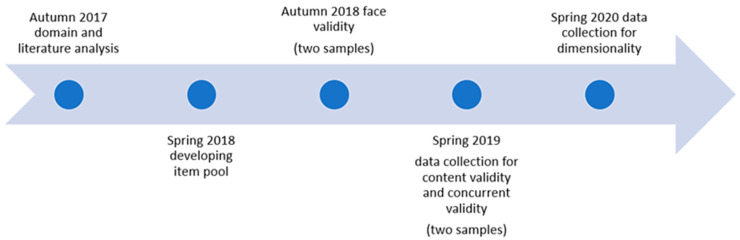
Overview of the scale development and validation processes.

All participants were informed about the study’s aim and procedures and that confidentially would be guaranteed. The participating nurses gave their informed consent for participation. The research was conducted in accordance with applicable ethical rules and national laws, and the research plan for the web-based surveys was approved by the Swedish Ethical Review Authority. An overview of the development of the scale and main validation processes is depicted in Figure 1.

### 2.1. Devising the Scale

The three dimensions of self-efficacy [12]—generality, magnitude, and strength—guided the devising process. A domain analysis was conducted prior to the creation of the items [13]. As a basis for the items, we used the latest available evidence-based guidelines for preventing healthcare-associated infections [1]. To capture the dimension of generality, items were developed to assess perceived self-efficacy in five characteristic interventions associated with nurses’ infection prevention behaviours: hospital environmental hygiene, hand hygiene, use of personal protective equipment, safe use and disposal of sharps, and principles of asepsis. Since beliefs in self-efficacy may vary in magnitude relative to situational demands, the pool of items assess some related situations in which the infection prevention measures vary in difficulty. An 11-point unipolar response scale ranging from 0 (not sure at all) to 10 (totally sure) was applied, as this was considered a simpler response format than the standard 100-point self-efficacy scale divided into 10-unit intervals [13]. Summing the item ratings into a total score gives a general overall estimate of the perceived strength of the beliefs in self-efficacy in regard to the practice of medical asepsis in care situations.

### 2.2. Validation Process

#### 2.2.1. Face Validity

Cognitive debriefing discussions were conducted with a total of five registered nurses and licensed practical nurses to evaluate the applicability, relevance, and comprehensiveness of the devised scale. These nurses were recruited from one medical ward in a county hospital. All gave informed consent to participate. They were asked to complete the scale in the presence of a research assistant who made notes of any observed difficulties or hesitations. Participants were then invited to comment on the items in the scale and the instructions, i.e., whether they found the items and instructions relevant and satisfactory, and if any important aspects had been omitted. As a result, some proposed items were deleted since they were not generally applicable to all nurses. Additionally, linguistic adjustments were made to ease the understanding of items. To ensure the understanding of the revised items, tests were performed with an additional five registered nurses, which resulted in a minor linguistic adjustment in the scale’s instructions. This adjustment made it clearer for respondents to judge their capability as “currently”.

#### 2.2.2. Content Validity

Content validity was estimated quantitatively by calculating the Content Validity Index (CVI) using a Microsoft Excel 365 spreadsheet. The CVI is an approach used to assess the content validity of individual items (I-CVI) and the overall scale (S-CVI) [14]. Ten infection prevention nurses were asked to indicate if they considered the content of each item as “not”, “somewhat”, “quite”, or “highly” relevant to how to practice medical asepsis in care situations. All gave informed consent to participate. I-CVI is expressed as the proportion of the infection prevention nurses that considered each item as “quite” or “highly” relevant, and S-CVI is calculated as the average of I-CVI across the items. I-CVI values of 0.78 or higher and a minimum S-CVI value of 0.90 were considered acceptable [14].

#### 2.2.3. Concurrent Validity

For assessing concurrent validity of the devised self-efficacy scale, registered nurses and licensed practical nurses (*n* = 106) were, by convenience sampling, recruited from a total of eight medical wards located in two Swedish hospitals. The only inclusion criterion was that the nurses would be on duty at the time of the data collection. To collect the data, a web-based survey was sent to the nurses’ work e-mail addresses. Informed consent was given in the survey by checking a designated checkbox. Used alongside the devised self-efficacy scale was a self-report form for demographical data, and the 10-item General Self-Efficacy Scale (GSE) [15]. The GSE uses a 4-point Likert scale ranging from 1 (not at all true) to 4 (absolutely true). GSE item responses are summed and divided by 10 to form a scale score, where higher scores indicate a higher level of general self-efficacy. The validity and reliability of the GSE have been well-established [16,17]. In the present study, the GSE’s Cronbach’s alpha value was 0.86. For concurrent validity, Spearman correlation coefficient was calculated using IBM SPSS Statistics for Windows (Version 27.0. IBM Corp., Armonk, NY, USA) to confirm the correlation between the devised self-efficacy scale and the GSE. A correlation between these self-efficacy measures is expected, but it should not be high since the scales are intended to measure different aspects of self-efficacy. That is, the hypothesis is that there is a positive correlation of weak to moderate magnitude between the devised self-efficacy scale and GSE.

#### 2.2.4. Data Quality and Acceptability

Data completeness was evaluated by the percentage of items with missing responses, which should be less than 10%. Targeting, i.e., how well scale scores are in agreement with levels of self-efficacy, was assessed through score distributions, skewness, and floor/ceiling effects [18]. Data completeness was evaluated using IBM SPSS Statistics for Windows (Version 27.0. IBM Corp., Armonk, NY, USA). To eliminate inappropriate items before inclusion in the exploratory factor analysis, we used the Robust Measure of Sample Adequacy (MSA) using Factor version 12.01.02 (Rovira I Virgili University, Tarragona, Spain). Items showing MSA values below 0.50 were removed from the item pool, as endorsed by Lorenzo-Seva and Ferrando [19].

#### 2.2.5. Structural Validity

A sample of registered nurses and licensed practical nurses (*n* = 525) were recruited from medical, surgical, and orthopaedic wards located in 22 Swedish hospitals. The only inclusion criterion was that the nurses would be on duty at the time of data collection. For data collection, a web-based survey was sent to the nurses’ work e-mail addresses. Informed consent was given in the survey by checking a designated checkbox. Self-reported demographical data were also asked for in the survey.

Exploratory factor analysis (EFA) was used to evaluate the dimensionality of the devised self-efficacy scale. Classical test theory using Factor version 12.01.02 was used to perform the analysis (Rovira I Virgili University, Tarragona, Spain). Due to the ordinal nature of item-level data [20], we applied a polychoric correlation matrix in the EFA with minimum rank factor analysis as an extraction method. As the sample was larger than 400 observations, and the Salomon method that splits the sample into two equivalent halves was applied [21]. The Ratio Communality Index (= 0.981) showed an optimal split and representativeness. The Kaiser–Meyer–Olkin test was good, with a value = 0.858: Bartlett’s test of sphericity = 3094.5, *p* < 0.0001. A parallel analysis based on 500 random permutations was used to decide the number of factors, since the otherwise commonly used Kaiser criterion (retain factors with eigenvalues greater than one) is known to yield biased results [22].

## 3. Results

Sample characteristics are described in Table 1. Most respondents were women (90.5%). 

### 3.1. Face and Content Validity

**Figure 2:**
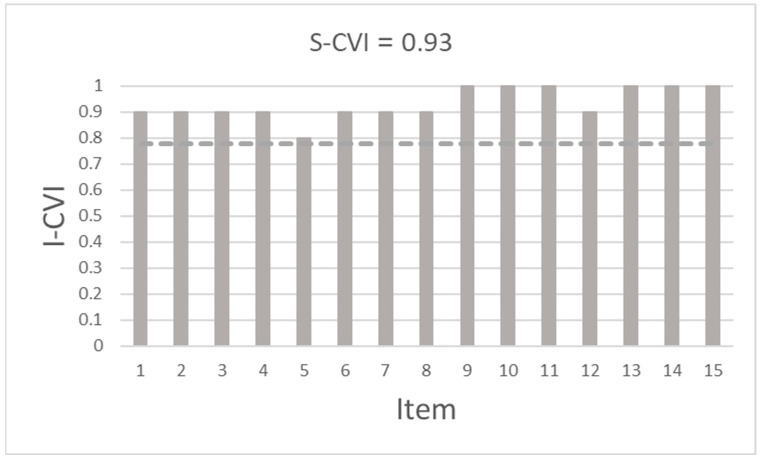
Content Validity Index of individual items (I-CVI) and the overall scale (S-CVI).

It was concluded from the face validity tests that the instructions and items were easy to understand and respond to with the 0−10 response format. The item relevance ratings for calculation of Content Validity Index resulted in I-CVI and S-CVI values above the respective threshold (Figure 2). Thus, the 15 devised self-efficacy items were assessed as adequately capturing the nurses’ beliefs in their abilities to practice medical asepsis in care situations.

### 3.2. Item Descriptive Statistics

Data completeness was very good. Missing data varied between 0 and 1.7% across the devised self-efficacy items. Three items had no missing values (Table 2). Of all respondents, 93.1% had no missing data, 6.1% had one missing item, and 0.8% had two items with missing data. Targeting assessments found evidence of ceiling effects for all items, but no floor effect was seen. For 9 of the 15 items, scores spanned the full possible range. Item 6 demonstrated an extremely skewed to the left score distribution with an almost complete ceiling effect. Item-level endorsement frequencies of the devised self-efficacy scale are reported in Table 2. The Measure of Sample Adequacy (MSA) values were above the threshold for all items except for one (Table 2). Consequently, item 6 was excluded from the item pool and further analyses. The average total self-efficacy score (126.6, 95%CI 125.4–127.6) for the devised 14-item scale was clearly above the scale midpoint with scores that ranged between 71 and 140. The skewness was −1.6.

### 3.3. Concurrent Validity and Structural Validity

As hypothesised, the correlation between the devised self-efficacy scale and the General Self-Efficacy Scale was weak (rs = 0.266, *p* = 0.006), and this finding supports the external construct validity. The results from the parallel analysis supported a unidimensional model. Eigenvalues for the first and second empirical factor were 5.97 and 1.14, respectively. The one-factor model explained 58.6% of the total variance. The factor loadings ranged between 0.365 and 0.789 (Table 3). The internal consistency using Cronbach’s alpha was 0.83.

## 4. Discussion

The purpose of this methodological paper was to report on the development and validation of a scale measuring nurses’ beliefs in self-efficacy in regard to their abilities to practice medical asepsis in inpatient care situations: the Infection Prevention Appraisal Scale (IPAS). Infection prevention is a vital issue for every healthcare setting. Even so, risk behaviours for organism transmission occur frequently in practice [2,3,4,5,6]. Self-efficacy has been identified as a commonly omitted determinant of behaviour change in infection prevention improvement strategies [23]. One possible reason for this is the identified lack of properly validated instruments measuring self-efficacy in regard to medical asepsis among healthcare personnel [11]. The present findings indicate that the 14-item IPAS is a unidimensional, reliable, and partially valid scale that can be used to assess nurses’ sense of self-efficacy to practice medical asepsis in inpatient settings. Thus, the development of IPAS might open future scenarios for additional improvement strategies in infection prevention, both in research and clinical practice.

IPAS (15 items) was found to be comprehensive and easy to understand in the conducted face validity tests, and item content was endorsed by infection prevention nurses in the content validity test. Since perceptions of self-efficacy are judgements of capability, it is of utmost importance that the items included are understood in terms of “can do”, otherwise they would not reflect the construct of interest [13]. However, 1 of the 15 developed items, despite adequate face and content validity evaluations, was excluded from the scale because it was evidently an inappropriate item that did not contribute to the overall adequacy of the scale [19]. The situation reflected by the excluded item was simply so easy to achieve that it did not contribute to variation in the scale and thus was unnecessary to measure. One lesson from this is that the inclusion of more participants or additional groups from the target population in the face-validity test might have enabled earlier detection of this shortcoming. Securing content validity is considered a critical step in scale development [14], and for self-efficacy scales, it is essential to include items accurately so that they reflect the construct of interest and are well-distinguished from related constructs [13]. With this limitation in mind, one could question why we only included infection prevention nurses as experts in the content validity evaluation, especially since we did not have information about their knowledge in self-efficacy theory constructs. However, in our defence, one of the researchers is very familiar with the theory and has experience in developing/translating self-efficacy scales (e.g., [24,25]). Even though this does not outweigh the limitations of the recruited expert sample, theoretically appropriate items devised for the scale have likely been secured.

Concurrent validity of IPAS was tested as an estimate of construct validity. The positive correlation between the devised scale and the General Self-Efficacy Scale was expected by underlying theory because both scales measure self-efficacy. Nevertheless, the General Self-Efficacy Scale refers to everyday life and should therefore not correlate highly with the situational demands and circumstances reflected in the devised scale. There are also empirical findings that support an expected positive weak correlation between general and situation-specific self-efficacy scales [24].

A well-targeted scale, according to classical test theory, should have an average score close to the scale midpoint and span most of its potential range without excess skewness (preferably between −1 and 1), and the floor/ceiling effects should not exceed 20% [18]. However, from a theoretical perspective, self-efficacy scales do not have quite the same measurement conditions as other concepts, because it is not reasonable that a normal distribution of total scale scores exists. That is, summed up from several items, zero or very low estimates of situation-specific self-efficacy are not to be expected, while to some extent, these estimates should be seen for individual items. It is actually described in the literature that self-efficacy scales are expected to show negatively skewed ratings [25,26]. Nonetheless, an obvious ceiling effect was present, which might impact the responsiveness and the interpretability of the IPAS. It also leaves limited room for detecting improvements in intervention studies if IPAS is used as an outcome measure. However, when looking at summed scores, it appears that to some degree, IPAS will be able to detect an improvement in self-efficacy

Based on these findings, the previous void of being able to measure self-efficacy in regard to infection prevention measures is now, to some extent, filled. By using the IPAS in quality improvement projects and research, nurses’ conceptions of their abilities to practice medical asepsis in inpatient care situations can be captured. This appraisal scale could be of assistance when attempting to improve adherence to infection prevention measures. The scale can be used for screening and as a tool in process evaluations, but its structural validity (by confirmatory factor analysis) and predictive validity need to be tested in new samples before the scale is applied as an outcome measure in intervention studies that focus on beliefs in self-efficacy. The Infection Prevention Appraisal Scale (IPAS) consists of 14 items covering characteristic interventions associated with nurses’ infection prevention behaviour. The items reflect five aspects of medical asepsis as well as general and specific hygiene principles. These aspects are aseptic technique (3 items), disinfection (4 items), glove usage (3 items), fingernails (1 items), and work clothes (3 items).

### Limitations

There are several limitations of this methodological study that need to be considered. The empirical parts may have affected the development of items as well as the interpretation of results because there could be a selection bias due to the convenience sampling procedure. The relatively small sample size used for concurrent validity poses a risk of potential bias. However, based on available demographic data, the sample can be considered representative of the population. Another limitation is that the scale was developed and tested in the Swedish healthcare setting. Socio-cultural validation is needed because items may not be appropriate in other settings.

## 5. Conclusions

In conclusion, the Infection Prevention Appraisal Scale demonstrates sound psychometric properties supporting a unidimensional measure of self-efficacy to medical asepsis in inpatient care situations.

## Figures and Tables

**Table 1:** Demographic characteristics of the samples.

Characteristic	Sample, Structural Validity (*n* = 525)	Sample, Concurrent Validity (*n* = 106)
Gender	*n* (%)	*n* (%)
Women	475 (90.5)	95 (90.5)
Men	45 (8.6)	8 (7.6)
Other	2 (0.4)	2 (1.9)
Missing value for gender	3 (0.6)	1 (0.9)
	Mean (SD)	Mean (SD)
Age, years	40.8 (12.8)	41.8 (11.8)
Working as a nurse, years	14.0 (12.8)	14.8 (12.2)

**Table 2:** Item endorsement frequencies of the Infection Prevention Appraisal Scale (IPAS), *n* = 525.

Item Score Distribution, *n* (%)													
Item	MSA	0	1	2	3	4	5	6	7	8	9	10	Missing
1	0.93	4 (0.8)	1 (0.2)	4 (0.8)	9 (1.7)	7 (1.3)	32 (6.1)	9 (1.7)	30 (5.7)	81 (15.4)	93 (17.7)	254 (48.4)	1 (0.2)
2	0.86	1 (0.2)	1 (0.2)	2 (0.4)	4 (0.8)	-	10 (1.9)	4 (0.8)	9 (1.7)	23 (4.4)	31 (5.9)	440 (83.8)	-
3	0.90	-	-	1 (0.2)	1 (0.2)	3 (0.6)	24 (4.6)	15 (2.9)	32 (6.1)	90 (17.1)	128 (24.4)	227 (43.2)	4 (0.8)
4	0.92	3 (0.6)	-	3 (0.6)	6 (1.1)	10 (1.9)	19 (3.6)	12 (2.3)	29 (5.5)	54 (10.3)	105 (20.0)	282 (53.7)	2 (0.4)
5	0.87	4 (0.8)	3 (0.6)	7 (1.3)	9 (1.7)	12 (2.3)	40 (7.6)	26 (5.0)	40 (7.6)	76 (14.5)	95 (18.1)	211 (40.2)	2 (0.4)
6 ^a^	0.36	-	-	-	-	-	-	-	1 (0.2)	4 (0.8)	4 (0.8)	516 (98.3)	-
7	0.59	-	-	1 (0.2)	2 (0.4)	-	4 (0.8)	2 (0.4)	6 (1.1)	11 (2.1)	22 (4.2)	474 (90.3)	3 (0.6)
8	0.90	-	-	1 (0.2)	4 (0.8)	1 (0.2)	4 (0.8)	2 (0.4)	11 (2.1)	46 (8.8)	77 (14.7)	378 (72.0)	1 (0.2)
9	0.96	1 (0.2)	1 (0.2)	3 (0.6)	5 (1.0)	1 (0.2)	6 (1.1)	12 (2.3)	30 (5.7)	73 (13.9)	117 (22.3)	276 (52.6)	-
10	0.80	-	-	1 (0.2)	1 (0.2)	3 (0.6)	6 (1.1)	1 (0.2)	4 (0.8)	27 (5.1)	70 (13.3)	409 (77.9)	3 (0.6)
11	0.91	2 (0.4)	-	3 (0.6)	6 (1.1)	8 (1.5)	14 (2.7)	14 (2.7)	33 (6.3)	83 (15.8)	107 (20.4)	246 (46.9)	9 (1.7)
12	0.88	1 (0.2)	-	-	2 (0.4)	-	2 (0.4)	1 (0.2)	8 (1.5)	9 (1.7)	34 (6.5)	466 (88.8)	2 (0.4)
13	0.74	13 (2.5)	9 (1.7)	18 (3.4)	19 (3.6)	11 (2.1)	28 (5.3)	9 (1.7)	17 (3.2)	43 (8.2)	48 (9.1)	305 (58.1)	5 (1.0)
14	0.86	-	2 (0.4)	3 (0.6)	5 (1.0)	3 (0.6)	21 (4.0)	12 (2.3)	21 (4.0)	64 (12.2)	94 (17.9)	297 (56.6)	3 (0.6)
15	0.85	1 (0.2)	1 (0.2)	1 (0.2)	1 (0.2)	2 (0.4)	7 (1.3)	6 (1.1)	14 (2.7)	37 (7.0)	89 (17.0)	361 (68.8)	5 (1.0)

MSA = Measure of Sample Adequacy; a = Item removed before exploratory factor analysis because of MSA < 0.50.

**Table 3:** Exploratory factor analysis of the 14-itema Infection Prevention Appraisal Scale.

Item *	Factor Loadings	Communality Values
1	0.588	0.515
2	0.612	0.727
3	0.789	0.844
4	0.787	0.745
5	0.675	0.677
7	0.365	0.683
8	0.688	0.720
9	0.677	0.513
10	0.438	0.461
11	0.733	0.764
12	0.546	0.597
13	0.365	0.720
14	0.698	0.925
15	0.749	0.829

* = The developed item 6 was excluded before exploratory factor analysis because the value on the Measure of Sample Adequacy test was too low.

## Data Availability

The participants of this study did not give written consent for their data to be shared publicly, so due to legal restrictions data is not publicly available.
